# Evaluation of disease activity in a population of Russian JIA patients

**DOI:** 10.1186/1546-0096-9-S1-P209

**Published:** 2011-09-14

**Authors:** OM Kostareva, IP Nikishina, SF Erdes, SO Salugina

**Affiliations:** 1 Scientific research Institute of Rheumatology of RAMS, Moscow, Russian Federation

## Background

The estimation of activity of disease in JIA patients is difficult, and the instruments of an estimation borrowed from adult rheumatology aren`t much useful for JIA patients.

## Objectives

To assess the correlation between disease activity scores, using the Juvenile Arthritis Disease Activity Score (JADAS), Disease activity score 28 (DAS 28) and Physician`s assessment of disease activity by VAS (PhGloVAS) on a cohort of JIA patients including in the cross-sectional epidemiological study in Russian Federation (RUS).

## Methods

During 3-moths cross-sectional study in 30 centers of RUS the data of 1099 consecutive patients were collected. The following key determinants of disease activity were examined: numbers of tender, swollen joints; number of active joint and joints with patient assessment of pain and global health scores (VAS); ESR; DAS 28; JADAS-71, PhGloVAS. Relationship between studied variables was investigated using of a nonparametric method of Spearman's correlation analysis.

## Results

There were 1099 patients with a mean age of 10.51 ± 4.54 years and the female/male ratio was 1.6. The distributions of JIA patients according to ILAR cacategories were as follows: systemic 122 (11.1%), oligoarticular 483 (43.94%), RF positive polyarthritis 81 (7.37%), RF negative polyarthritis 350 (31.84%), enthesitis-related 45 (4.09%), psoriatic 18(1.63%). The median disease duration was 4.5±3.85 years. The mean value of JADAS-71 score was 13.04±10.46 (95% CI 0.618), the mean value of DAS-28 score was 3.13±1.46 (95% CI 0.08) and mean value PhGloVAS scales was 37±24(95% CI 1.0). The JADAS-71 correlated with PhGloVAS (r=0.8, p<0.05) and DAS 28 (r=0.79, p<0.05). Patients have been distributed on groups according to degree of activity depending on index DAS 28. In group with remission (DAS 28<2.6) were 445 patients (40.49%), with low activity (DAS 28 2.6-3.2) were 208 patients (18.93%), with moderate activity (DAS 28 3.2-5.1) were 343 patients (31.21%), with high activity (DAS 28 >5.1) were 103 patients (9.37%). In each group the correlation between JADAS, DAS 28 was defined. The strongest relationship was determined in patients with high activity (r=0.79-0.68, p<0.02), and the poor relationship was in group with low activity (r=0.79-0.21, p<0.0001). In this group (n= 208) percent of the tendered and swelled joints which are not considered in an index DAS 28, were 51,1% and 40,87% respectively. The greatest percent of not considered involved joints was in group of patients with a sJIA (68.9% tender joints and 71.23% swelled). Hip, talocrural, and temporal-mandibular joints often enough are involved at this diagnosis (18.03%, 44.26%, 2.46% respectively in our analysis) and define functional insufficiency. As a whole, on all cohort of patients the poor relationship between DAS and JADAS is at patients with enthesitis-related arthritis ((r=0.79-0.63, p<0.036).

## Conclusion

JADAS and DAS 28 demonstrated strong correlation at a high degree of activity and poor correlation at a low degree of activity. Joints of the bottom extremities and others aren't considered in index DAS 28, but make appreciable percent from total number of the involved joints . DAS 28 it is applicable for an estimation of activity of disease not at all diagnoses JIA

